# Enhanced antibacterial activity of capped zinc oxide nanoparticles: A step towards the control of clinical bovine mastitis

**DOI:** 10.14202/vetworld.2019.1225-1232

**Published:** 2019-08-11

**Authors:** H. F. Hozyen, E. S. Ibrahim, E. A. Khairy, S. I. El-Dek

**Affiliations:** 1 Department of Animal Reproduction and AI, National Research Centre, Dokki, Giza, Egypt; 2 Department of Microbiology and Immunology, National Research Centre, Dokki, Giza, Egypt; 3 Materials Science and Nanotechnology Department, Faculty of Postgraduate Studies for Advanced Sciences, Beni-Suef University, Beni-Suef, Egypt

**Keywords:** antibacterial activity, clinical mastitis, dairy cows, zinc oxide nanoparticles

## Abstract

**Background and Aim:**

Bovine mastitis is the costliest prevalent disease in the dairy sector due to the limitations of conventional treatments. Zinc oxide nanoparticles (ZnO-NPs) have been regarded as safe and economical antibacterial candidates against several microorganisms, but the tendency of these particles to aggregate is a major barrier to their application. This study aimed to enhance the antibacterial efficiency of ZnO-NPs against some bacterial agents, causing bovine mastitis.

**Materials and Methods:**

A total of 24 milk samples out of 300 cases from Nubaria farm, Beheira Governorate, Egypt, were collected from cows with clinical mastitis. ZnO-NPs were fabricated by a sonochemical method using starch as a capping agent and by an auto-combustion reaction using glycine as a fuel. The two preparations of synthesized ZnO-NPs at different concentrations were assessed for their antimicrobial activities *in vitro* against *Staphylococcus aureus*, *Escherichia coli*, and *Klebsiella pneumoniae* isolated from milk of affected cows.

**Results:**

Sonochemically synthesized capped ZnO-NPs were dispersed and non-agglomerated in comparison with aggregated uncapped ZnO-NPs prepared by an auto-combustion reaction. Capped dispersed ZnO-NPs showed higher antibacterial activity against *S. aureus*, *E. coli*, and *K. pneumoniae* than particles synthesized by the auto-combustion reaction at same concentrations. However, the zone of inhibition for dispersed and agglomerated ZnO-NPs was concentration-dependent. In addition, Gram-positive *S. aureus* exhibited higher resistance to ZnO-NPs synthesized by both methods than Gram-negative *E. coli* and *K. pneumoniae*.

**Conclusion:**

Dispersed, non-agglomerated ZnO-NPs fabricated using starch as a capping agent under sonochemical irradiation could potentially be regarded as highly effective and inexpensive antimicrobial agents against *S. aureus*, *E. coli*, and *K. pneumoniae* for the management of bovine mastitis.

## Introduction

Bovine mastitis causes severe economic loss to the dairy industry [1] and poses considerable risks to public health because the major species of bacterial pathogens causing mastitis are toxin producers that can contaminate milk [2]. Moreover, mastitis could have harmful effects on the ovarian follicular response and lower conception and fertility in cows [3]. Both clinical and subclinical mastitis can effectively impair oocyte competence, resulting in low production of blastocysts [4]. Several contagious and environmental pathogens have been reported to cause clinical and subclinical mastitis [5]. *Staphylococcus aureus* is one of the most important contagious microorganisms affecting the mammary glands of infected cows, and *Escherichia coli* and *Klebsiella* spp. are the main environmental mastitis-causing pathogens [6].

To date, bovine mastitis has been commonly treated with the administration of antibiotics [7]. This approach has many disadvantages, including increasing resistance to antibiotics, a low cure rate, and the presence of antibiotic residues in milk [8]. According to the World Health Organization report, the resistance of bacteria to antibiotics is a major global hazard to public health after global warming and terrorism [9]. Therefore, there is an urgent need to develop methods for the formulation of new, safe, and cost-effective antibiotics [10,11]. In this context, metal oxide nanoparticles have emerged as promising antibacterial materials [12]. Zinc oxide nanoparticles (ZnO-NPs) are among the most common oxides that have received significant interest worldwide in biological applications [13] and have been regarded as safe materials for animals and humans [14].

ZnO-NPs have been shown to have selective toxicity toward bacteria but exhibit minimal effects on human cells [15]. ZnO-NPs have been investigated as safe and economic antibacterial candidates against several microorganisms [16,17]. However, the tendency of ZnO-NPs to aggregate reduces the exposed surface area and limits the application of these particles [18] because aggregation of nanoparticles could prevent the interaction of the nanoparticles with bacterial cell walls, leading to inhibition of the antibacterial activity of the particles [19].

We hypothesized that increased strength of antibacterial activity against mastitis-causing pathogens could potentially be achieved using capped, dispersed, and non-agglomerated ZnO-NPs. To test this hypothesis, we synthesized ZnO-NPs through two different routes: The sonochemical route using starch as a capping agent to prevent agglomeration of ZnO-NPs and conventional auto-combustion reaction without capping agents. Antibacterial properties and dose effect of ZnO-NPs against *S. aureus*, *E. coli*, and *K. pneumoniae* isolated from milk of cows affected with clinical mastitis were evaluated.

## Materials and Methods

### Ethical approval

The animals during the experiment were handled in accordance with the use and animal care committee of National Research Center, Egypt.

### Animals

Three hundred lactating cows were inspected at the El-Nubaria farm, Beheira Governorate, Egypt, during the period from September 2017 to December 2017. The udders were examined and cows with clinical mastitis were identified through observation and palpation of the udders, clots in milk and mammary gland inflammation. Animals were not treated with any antibiotic for at least 30 days before sample collection.

### Milk samples

Twenty-four pooled milk samples were collected from 24 lactating cows affected with clinical mastitis and were used in this study. The udder teats were sterilized with 70% ethyl alcohol before collection of milk samples. 10 ml of milk was milked into sterilized tubes and samples were kept on ice during transportation to the laboratory. Bacteriological examination was performed within 2 h after sample collection.

### Synthesis of ZnO-NPs

Zinc nitrate (Sigma-Aldrich, India), starch (ADWIC, Egypt), sodium hydroxide pellets (Merck, Germany), glycine (Merck, Germany), and deionized water were used as conventionally.

Capped ZnO nanostructures were synthesized by sonochemical route according to the method of Luo *et al*. [20] with some modifications. Briefly, 0.3 M zinc nitrate was dissolved in 100 ml of cooled starch solution (1.5 g in 100 ml of distilled water) by a magnetic stirrer at 500 rpm for 10 min, and 0.6 M sodium hydroxide precursor was dissolved separately in 100 ml of distilled water by magnetic stirring at 500 rpm for 10 min and kept ready. Sodium hydroxide solution was added dropwise to the zinc nitrate hexahydrate solution containing starch under sonication using an ultrasonic probe (operating at a frequency of 20 kHz) for 30 min with 5 s pulse and 5 s off cycle. After complete addition of sodium hydroxide solution to zinc nitrate hexahydrate solution in the first 30 min, the whole mixture (zinc nitrate hexahydrate solution +starch+ sodium hydroxide) was again exposed to acoustic radiation by the same ultrasonic horn for additional 10 min. Particles were washed with distilled water followed by methanol. The final product was dried at 150°C for 12 h and then calcinated at 600°C for 6 h.

Uncapped ZnO particles were synthesized by auto-combustion reaction using glycine as fuel according to the method of Ahmed *et al*. [21]. This process involved mixing pure Analar metal nitrate with glycine. The nitrate-to-glycine ratio was calculated based on the total oxidizing components and reducing coefficients required for the stoichiometric balance to reach an equivalence ratio of unity. In this case, the energy released was maximum. The reactants were mixed well and heated on a magnetic stirrer. Thermal dehydration resulted in a highly viscous liquid. On further heating, the viscous liquid swelled and auto-ignited, to yield a voluminous powder. The reaction was very fast and produced a very fine, fluffy, and dry powder. This method is quite simple, fast, and inexpensive and uses an exothermic and self-sustaining chemical reaction between the desired metal salts and a suitable organic fuel. The striking feature of this method is that the heat required to sustain the chemical reaction is provided by the reaction itself and not by an external source.

### Characterization of ZnO-NPs

The dispersion and shape of ZnO-NPs were characterized by high-resolution transmission electron microscopy (HR-TEM) at an accelerating voltage of 200 kV. X-ray diffraction (XRD) patterns were recorded on a PANalytical (Empyrean) X-ray diffractometer using Cu Kα1 radiation (wavelength 1.5406 Å) at an acceleration voltage of 40 kV, a current of 30 mA, a scan angle range of 5–80°, and a scan step of 0.02°. The size distribution of particles was determined by Zetasizer nano-Zs90 (Malvern, UK).

### Isolation and identification of bacteria

10 µl of each milk sample was cultured on mannitol salt agar for isolation of *S. aureus* and on MacConkey agar and Levine’s eosin-methylene blue (EMB) agar for the isolation of Gram-negative bacteria. The cultures were incubated at 37°C for 24 h followed by Gram staining and biochemical tests (catalase, DNase, coagulase tests [22] as well as IMViC tests).

### Determination of ZnO-NPs antibacterial activity by the agar well diffusion test, and determination of minimum inhibitory concentration (MIC) and minimum bactericidal concentration (MBC) of ZnO-NPs

The antibacterial activity of the ZnO-NPs was determined using Mueller-Hinton agar (MH) culture medium (Oxoid) as described for the diffusion disc method that is commonly used for antibiotic susceptibility tests [23]. Freshly cultured bacteria were inoculated in nutrient broth, and turbidity was matched with McFarland standard at 0.5. The bacterial suspensions were inoculated and spread in Petri plates containing MH agar medium, and then wells that were 5 mm in diameter were placed on the inoculated test organisms and instilled with ZnO-NPs at different concentrations (1, 3, 5, 10, 20, 30, 40, and 50 mg/ml). The Petri dishes were incubated at 37°C for 24 h and the inhibition zones around the wells were measured to determine antimicrobial activity.

The microdilution method with 96-well microplates was used with a slight modification to determine the MIC [24]. Briefly, 96-well plates were prepared by the distribution of 95 μl of MH broth medium and 5 μl of bacterial inoculated in each well. Furthermore, 100 μl of each prepared solution of ZnO-NPs (1-50 mg/ml) was poured into the wells to examine the MIC of ZnO-NPs. Then, the microplates were incubated for 24 h at 37°C. The growth rate was determined by measuring the absorbance at a wavelength of 600 nm using a microplate reader. To test the bactericidal effect, we transferred the contents of each well (200 ml) into an Eppendorf tube with 2 ml of BHI medium, and the tube was then incubated at 37°C for 12 h. Finally, 20 ml of medium was withdrawn from the cultures in which no turbidity was observed, inoculated on BHI agar, and incubated at 37°C for 24 h. The concentrations of ZnO-NPs that have bactericidal effects were selected based on the absence of colonies on the agar plate. The MBC value was defined as the ZnO-NP concentration at which 100% of bacterial growth was inhibited compared to a positive control (no treatment with ZnO-NPs).

### Statistical analysis

All statistical analyses were performed using the Statistical Package for the Social Sciences 22.0 (IBM, USA) for windows. Statistical analysis for the inhibition zones and effect of both types of ZnO-NPs on tested bacterial species was carried out using one-way analysis of variance, and the significance was set at 0.05. Inhibition zone graph was presented using GraphPad Prism software (GraphPad, San Diego, USA).

## Results

### Characterization of ZnO-NPs

**Figure-1:**
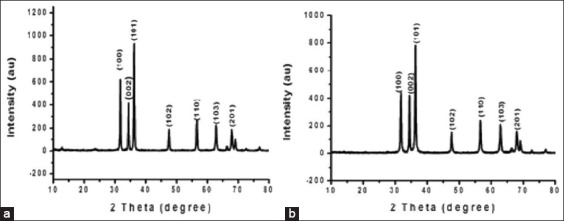
X-ray diffraction patterns of (a) starch capped zinc oxide nanoparticles (ZnO-NPs) prepared by sonochemical method and (b) uncapped ZnO-NPs prepared by auto-combustion reaction.

Figure-1a shows the XRD pattern of the capped ZnO-NPs synthesized by the sonochemical method. The peak characteristics of the sonochemically prepared ZnO-NPs were consistent with a face-centered cubic system. The main peaks at 2θ of 36.2492°, 31.7502°, and 34.4146° correspond to planes (101), (100), and (002), respectively. All peaks in the X-ray diffractogram can be easily indexed to a hexagonal cubic structure centered in the face of the zinc oxide according to the available literature (Joint Committee on powder diffraction standards), no (04-009-7657). For ZnO-NPs prepared by the auto-combustion reaction (Figure-1b), the XRD pattern also showed diffraction peaks at 2θ=36.3092°, 31.844°, and 34.4971°, which according to the JCPDS file no. 01-083-6338, correspond to the (101), (100), and (002) crystal planes of nano zinc oxide. The obtained XRD patterns confirm the formation of nano zinc oxide structures.

**Figure-2:**
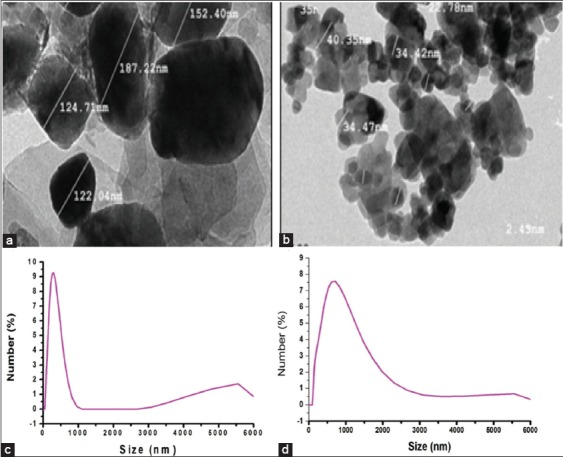
(a) High-resolution transmission electron microscopy (HR-TEM) images of starch capped zinc oxide nanoparticles (ZnO-NPs) prepared by sonochemical method, (b) HR-TEM images of uncapped ZnO-NPs prepared by auto-combustion reaction, (c) size-distribution of starch capped ZnO-NPs prepared by sonochemical method, and (d) size-distribution of uncapped ZnO-NPs prepared by auto-combustion reaction.

The microscopic analysis results obtained by HR-TEM presented in Figure-2a showed that the ZnO-NPs capped with starch were non-agglomerated and platelet shape of hexagonal form. The particle size exceeded 100 nm due to capping with high molecular-weight starch. On the other hand, HR-TEM images of the ZnO-NPs synthesized by the auto-combustion reaction showed hexagonal and aggregated particles with a size of approximately 41 nm (Figure-2b). The size distribution determined by Zetasizer was not wide for both capped and uncapped ZnO-NPs. The size distribution peak for starch capped (Figure-2c) and uncapped ZnO-NPs (Figure-2d) was about 174 nm and 669 nm, respectively.

### Isolation and identification of mastitis-causing bacteria

In this study, 8/24 samples showed yellow colonies on mannitol salt agar that was initially identified as *S. aureus*, which was confirmed by Gram staining, biochemical tests and catalase, and DNase and coagulase activity assays. On the other hand, 9/24 samples showed a metallic sheen on EMB agar, characteristic of *E. coli* spp., which was confirmed by biochemical identification using IMViC tests that showed positive results with both methyl red and indole. The remaining 7/24 samples showed opaque, mucoid colonies that were pink in color on MacConkey agar and exhibited urease activity, which is characteristic of *K. pneumoniae*, and were biochemically positive for citrate.

### Antibacterial activity of capped and non-capped ZnO-NPs against *S. aureus*, *E. coli*, and *K. pneumoniae*

**Figure-3:**
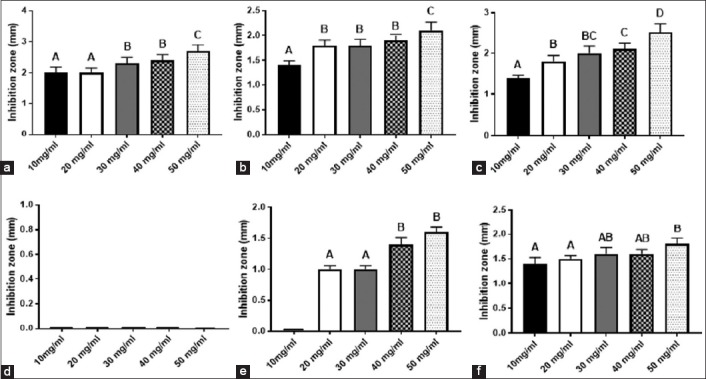
The size of the zones of inhibition of starch capped zinc oxide nanoparticles (ZnO-NPs) against (a) *Staphylococcus aureus*, (b) *Escherichia coli*, and (c) *Klebsiella pneumoniae* and uncapped ZnO-NPs against (d) *Staphylococcus aureus*, (e) *Escherichia coli* and (f) *K. pneumoniae*. Data are presented as mean ± standard deviation. One-way analysis of variance followed by Duncan multiple comparisons test (p<0.05).

The average diameters of the zones of inhibition (in mm) for capped and non-capped ZnO-NPs are presented in Figure-3. The presence of an inhibition zone clearly indicated the antibacterial effect of both types of ZnO-NPs except for non-capped ZnO-NPs with *S. aureus*. Capped ZnO-NPs showed high antibacterial activity against *S. aureus* and *K. pneumoniae* with the maximum zone of inhibition at a concentration of 50 mg/ml reaching 2.7 mm and 2.5 mm, respectively, followed by *E. coli* with a maximum zone of inhibition of 2.1 mm. On the other hand, non-capped ZnO-NPs were unable to inhibit *S. aureus* growth at all the tested concentrations (10, 20, 30, 40, and 50 mg/ml). However, these ZnO-NPs showed antibacterial effects against *E coli* and *K. pneumoniae* with maximum zones of inhibition of 1.6 and 1.8 mm, respectively. The zone of inhibition for both types of nanoparticles was concentration-dependent.

### MIC and MBC of both capped and non-capped ZnO-NPs against *S. aureus*, *E. coli*, and *K. pneumoniae*

Table-1 shows that MIC and MBC values for *S. aureus* were 20 mg/ml and 30 mg/ml, respectively, with capped ZnO-NPs. On the other hand, non-capped ZnO-NPs did not exert antibacterial effects on *S. aureus* at all the tested concentrations. The observed MIC for both capped and non-capped ZnO-NPs was 5 mg/ml with *E. coli* while the MBC was 10 mg/ml. In addition, the MIC and MBC values for *K. pneumoniae* were 5 mg/ml and 10 mg/ml, respectively, for non-capped ZnO-NPs. Capped ZnO-NPs showed bactericidal effects against *K. pneumoniae* at a concentration of 1 mg/ml.

**Table 1:** MIC and MBC of both capped and non-capped ZnO-NPs against *S. aureus*, *E. coli*, and *K. pneumoniae* isolated from milk of cows with clinical mastitis.

Bacterial strains	MIC		MBC	
	
	Capped ZnO-NPs	Uncapped ZnO-NPs	Capped ZnO-NPs	Uncapped ZnO-NPs
*S. aureus*	20 μl/ml	-	30 μl/ml	-
*E. coli*	5 μl/ml	5 μl/ml	10 μl/ml	5 μl/ml
*K. Pneumonia*e	<1 μl/ml	10 μl/ml	1 μl/ml	10 μl/ml

ZnO-NPs=Zinc oxide nanoparticles, MIC=Minimum inhibitory concentration, MBC=Minimum bactericidal concentration, *S. aureus*=*Staphylococcus aureus*, *E. coli=Escherichia coli*, *K. pneumoniae*=*Klebsiella pneumoniae*

## Discussion

Nanoparticles have been reported to have potential applications in the management of bovine mastitis infections [8]. The shapes and sizes of nanoparticles depend on the condition in which the nanoparticles are synthesized [25]. In the present study, the synthesis of ZnO-NPs using a sonochemical route produced platelet shape of the hexagonal form and well-dispersed nanoparticles, while ZnO-NPs synthesized by the auto-combustion reaction were hexagonal and agglomerated. Consistent with our results, it has been reported that the use of appropriate capping agents and sonication could prevent agglomeration and deposition of synthesized ZnO-NPs [26,27].

*S. aureus* is considered to be a major mastitis-causing pathogen due to its prevalence in dairy herds [28]. Moreover, the resistance of *S. aureus* to antimicrobial agents is a well-documented challenge in dairy cows [29]. In the present work, ZnO-NPs capped with starch showed antibacterial effects at concentrations of 1-50 mg/ml against *S. aureus* isolated from cows with clinical mastitis. In contrast, ZnO-NPs prepared by the auto-combustion method had no antibacterial effect against *S. aureus* at the same concentrations. The antibacterial activity of ZnO-NPs in our study was similar to that observed by Emami-Karvani and Chehrazi [30] by different preparation methods with different concentrations of ZnO-NPs. In this work, *E. coli* and *K. pneumoniae* were sensitive to ZnO-NPs. However, sonochemically capped ZnO-NPs exhibited higher antibacterial activity than ZnO-NPs synthesized by the auto-combustion reaction at the same concentrations and show larger zones of inhibition in both organisms. These results are consistent with those recorded by Danial and Yousef [31]. The low antibacterial activity of uncapped ZnO-NPs synthesized by the auto-combustion reaction due to the agglomeration of nanoparticles could be attributed to the fact that zinc oxide is nearly insoluble in water and agglomerates immediately with water during synthesis due to the high polarity of water, leading to deposition [32]. However, capping of the ZnO-NPs with starch, combined with sonochemical irradiation, facilitated the formation of well-dispersed and stable ZnO-NPs. The concentration of nanoparticles is an important factor affecting the antimicrobial activity of these particles [33]. In addition, Wahab *et al*. [34] reported that the growth inhibition increased with increasing concentrations of ZnO-NPs in well and discs. The concentration of nanoparticles affects toxicity directly, and at high concentrations of nanoparticles can release large amounts of ions [35,36].

The MBC and MIC values in the current study reflect that nano zinc oxide was more effective against Gram-negative *E. coli* and *K. pneumoniae* than Gram-positive *S. aureus*. It is hypothesized that the differing cell wall structures of both Gram-positive and Gram-negative cells are the reason for this phenomenon [19]. Many studies have found that Gram-positive bacteria are more resistant to the mechanisms of action of nanoparticles [26,27,30,37,38]. The cell wall in Gram-negative bacteria, such as *E. coli* and *K. pneumonia*e, is covered by a thin peptidoglycan layer with an additional outer lipopolysaccharide membrane. This arrangement may facilitate the entry of released ions from nanoparticles into the cell. However, Gram-positive bacteria are covered with very thick peptidoglycan with covalently attached teichoic and teichuronic acids, which restricts the entry of nanoparticles and acts as a protective layer. In contrast to our results, Paredes *et al*. [24] found that Gram-positive *S. aureus* was more sensitive to silver nanoparticles (AgNPs) than Gram-negative *E. coli* at the same AgNPs concentrations. The results of the present study are consistent with those of Ruparelia *et al*. [39] who found that Gram-positive *S. aureus* is more resistant to AgNPs than Gram-negative *E. coli*.

There is a debate regarding bacterial inhibition by ZnO-NPs and the underlying mechanisms of action. The antimicrobial properties of ZnO-NPs might be associated with the small size of these particles, which are 250 times smaller than a bacterium. As a result, it is easy for nanoparticles to adhere to the bacterial cell wall, causing destruction and death of bacteria [40]. Furthermore, it has been suggested that small nanoparticles have high surface reactivity and can be easily internalized by cells, and releasing Zn^+^, which could be toxic to the biomolecules in bacterial cells [41]. When metal ions in solution are exposed to bacterial cells, these ions become uniformly distributed in the environment surrounding the bacterial cell with no specific localization. In contrast, nanoparticles that interact with the bacterial cell wall produce a focal source that continuously releases ions, causing more toxicity to the cells [42]. The high ion concentration generated further helps cellular penetration. Therefore, nanoparticle dissolution is localized around the bacterial cell membrane, with the kinetics of dissolution depending on the size and shape of the nanoparticle [43]. Another possible explanation is that ZnO-NPs may induce the production of reactive oxygen species and cause membrane dysfunction [44]. Although the multiple pathways that seem to be simultaneously activated by nanoparticles make elucidation a difficult task, these pathways also the reason for the high efficacy of nanoparticle exposure. Apparently, this combination itself causes toxicity; it is unlikely that a single factor is responsible for the bacterial killing. The multi-target activity of nanoparticles is ideal for treatment for infections and destructions of pathogens [19].

## Conclusion

The results of the current work suggest that (1) ZnO-NPs as a new and cost-effective material exhibit strong antibacterial properties against *S. aureus*, *E. coli*, and *K. pneumoniae* isolated from milk samples obtained from the udders of cows with clinical mastitis. (2) Different synthesis methods and capping agents could influence the agglomeration, particle size, and morphology of fabricated ZnO-NPs and could consequently affect the antibacterial properties of these particles. (3) The synthesis of ZnO-NPs by a sonochemical method accompanied by capping with starch prevented agglomeration of ZnO-NPs and enhanced antibacterial activity of nanosized zinc oxide against the pathogen bacteria *S. aureus*, *E. coli*, and *K. pneumoniae* which cause bovine clinical mastitis.

## Authors’ Contributions

HFH and ESI designed the experiment. HFH collected samples. ESI and EAK evaluated the antibacterial activity of the synthesized nanoparticles. HFH and SIE synthesized and characterized the nanoparticles. HFH, ESI, and EAK drafted the manuscript. SIE helped draft the manuscript. HFH and ESI reviewed the manuscript efficiently. All authors read and approved the final manuscript.
